# Supplementary material for: Assessment of Immunogenicity and Efficacy of CV0501 mRNA-Based Omicron COVID-19 Vaccination in Small Animal Models
Source: Vaccines (Basel). 2023 Jan 31;11(2):318. doi: 10.3390/vaccines11020318 (PMC9965737; doi:10.3390/vaccines11020318)
Supplement: Supplementary file 1 [file vaccines-11-00318-s001.zip › vaccines-2173903-supplementary.pdf]

Supplementary Data

Supplementary Figure S1: CV0501 vaccine induced strong cross-nAbs at Day 42 post-immunization

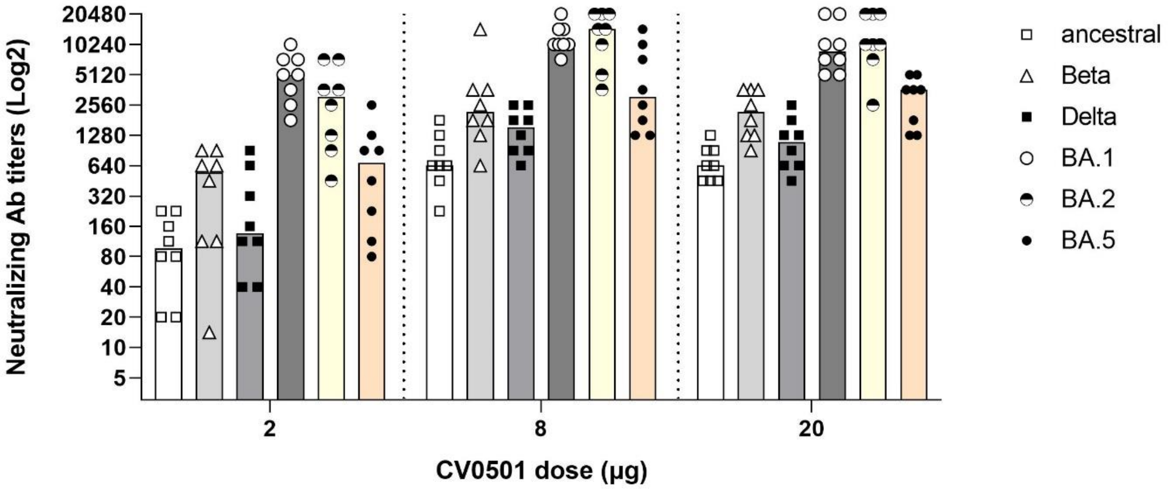

Figure S1: CV0501 vaccine induced strong cross-nAbs at Day 42 post-immunization (Data from Figures 1 and 2 displayed).

Wistar rats (n=8/group) were immunized i.m. on Days 0 and 21 with 2, 8 or 20 µg CV0501. nAbs against ancestral, Beta, Delta, BA.2 and BA.5 SARS-CoV-2 were assessed from serum isolated on Day 42 post-immunization. Each symbol represents an individual animal and bars depict the median value.

Supplementary Figure S2: CV0501 induces dose dependent IFN-γ secreting T cells

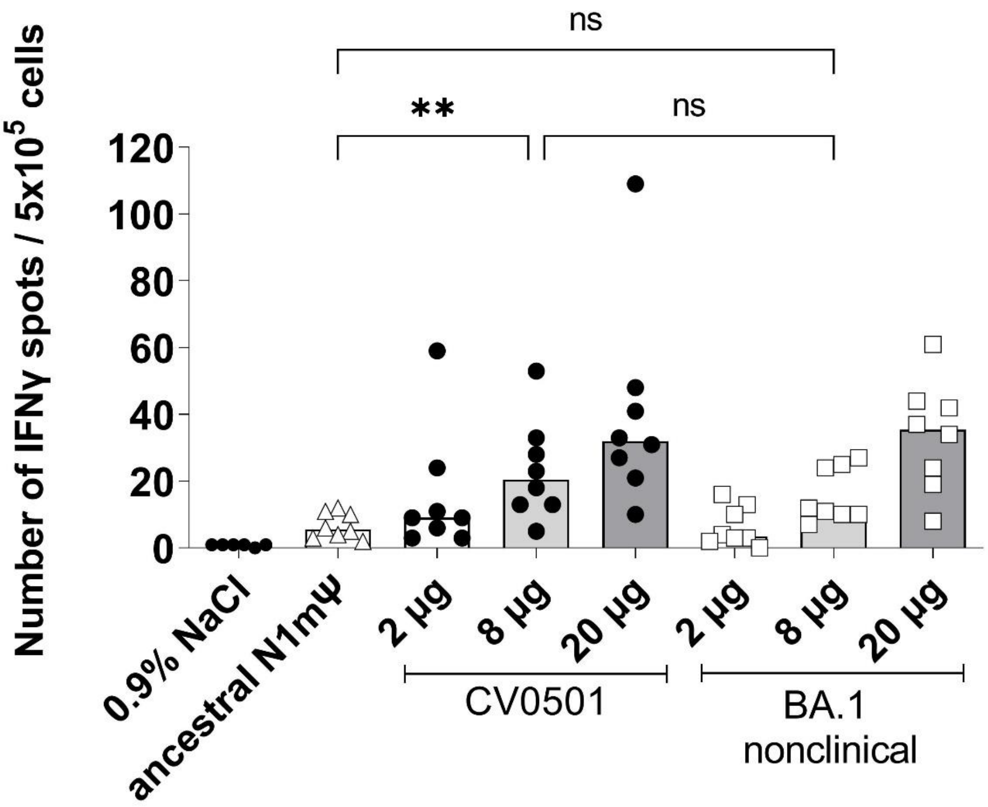

Figure S2: Immunization with CV0501 induced significantly higher IFN-γ secreting T cells compared with ancestral N1mΨ.

Splenocytes from rats immunized on Days 0 and 21 with either CV0501 (2, 8 or 20 µg), BA.1 nonclinical (2, 8 or 20 µg), ancestral N1mΨ (8 µg), or 0.9% NaCl buffer (sham control) were isolated on Day 42 and single-cell suspensions were prepared and stimulated for 24 hours at 37°C using a SARS-CoV-2 Omicron peptide library (JPT, PM-SARS2-SMUT08-1) at 1 µg/mL. Each dot represents an individual animal and bars depict the mean. Statistical analysis was performed using ANOVA and Dunn's multiple comparison test (**; p=0.0049).

Supplementary Figure S3: CV0501 booster immunization induces high cross-neutralizing antibodies against BA.1 in Wistar rats

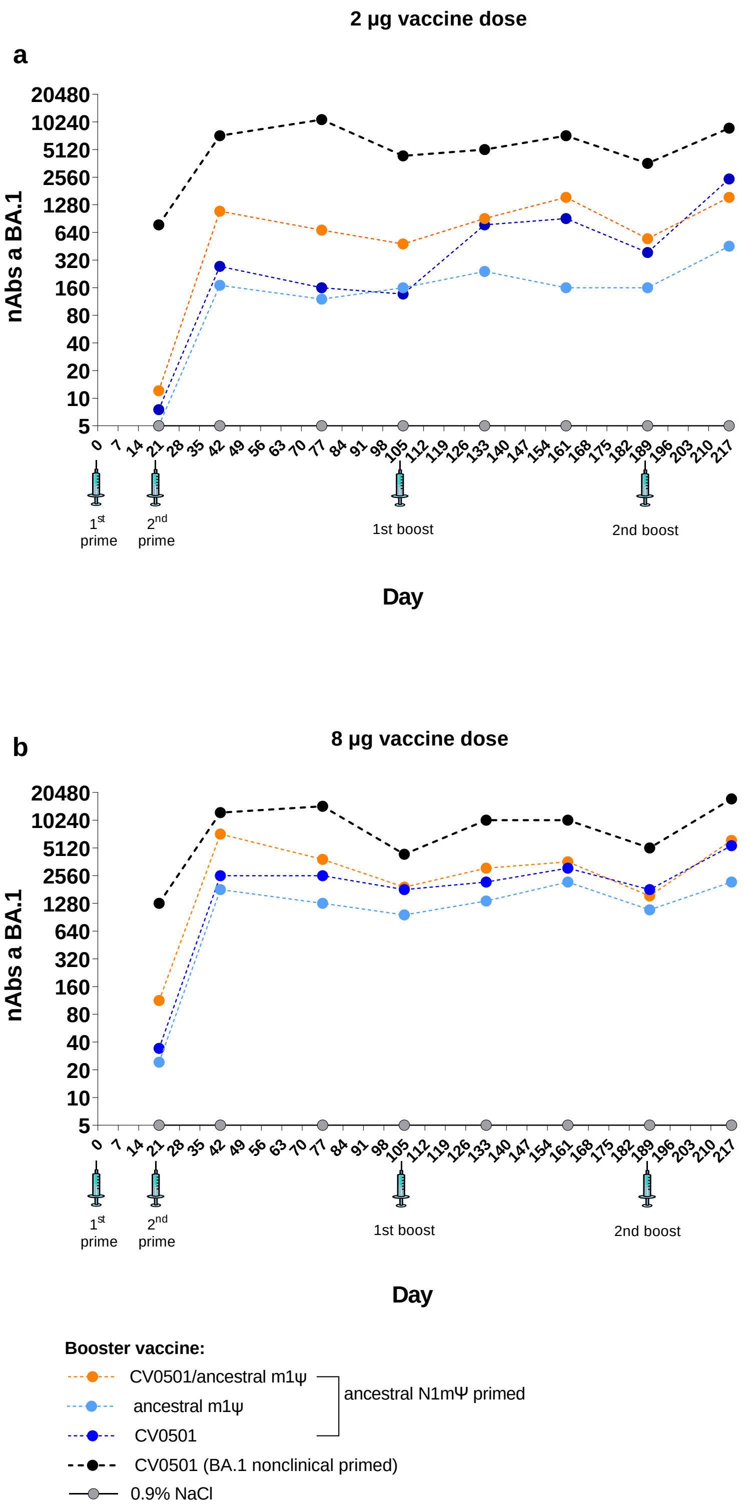

Figure S3: CV0501 booster immunization induces high cross-neutralizing antibodies against BA.1 in Wistar rats

Wister rats (n=8/group) were immunized on Days 0 and 21 with either 2 µg (**a**) or 8 µg (**b**) ancestral N1mΨ or BA.1 nonclinical. On Days 105 and 189, rats were given third and fourth (booster) doses of either CV0501 alone, ancestral N1mΨ alone, or bivalent CV0501/ancestral N1mΨ (half doses of each) at 2 µg or 8 µg doses. 0.9% NaCl was used as a sham control and was administered at the same timepoints. nAbs against BA.1 SARS-CoV-2 were assessed in serum obtained on Days 21, 42, 77, 105, 133, 161, 189 and 217 and analyzed at each timepoint. Each symbol represents the median value.

Supplementary Figure S4: Induction of nAb against BA.1 Omicron variants observed after CV0501 immunization.

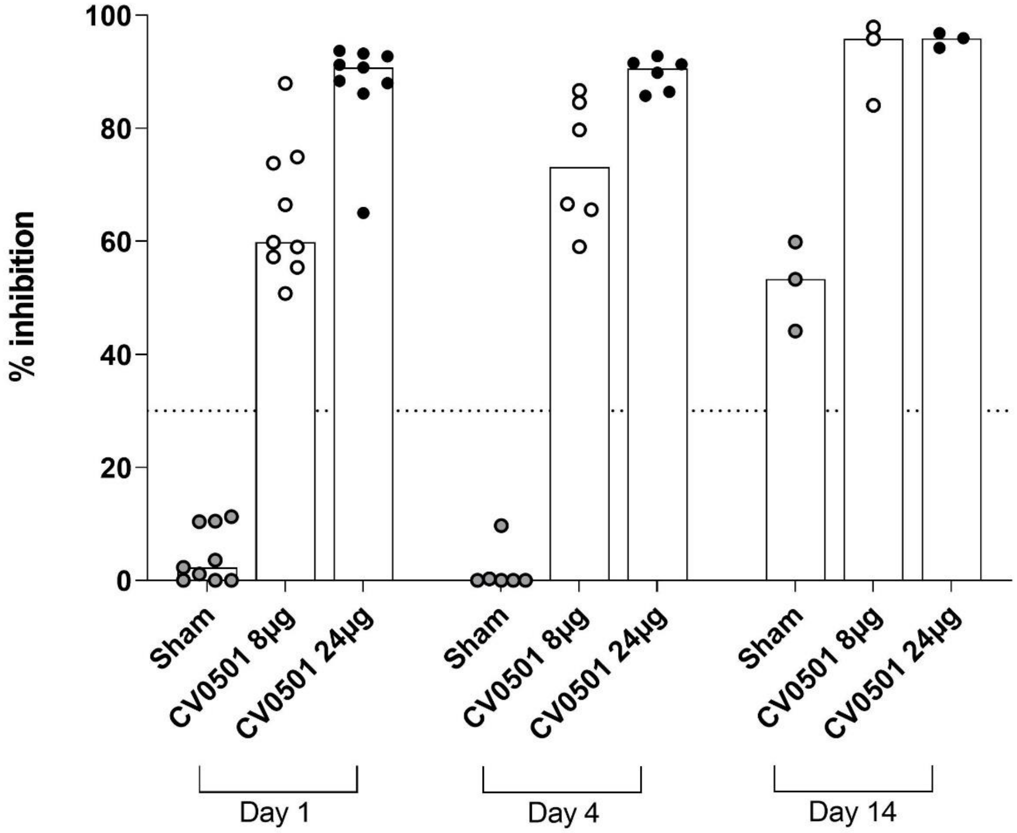

Figure S4: Percentage of inhibition against BA.1 Omicron variants observed after CV0501 immunization.

Hamsters (n=9/group) were immunized on Days 0 and 28 with 8 µg or 24 µg doses of CV0501 or 0.9% NaCl (sham controls) and challenged on Day 56 with Omicron BA.2 (10^5 $TCID_{50}$/animal administered i.n. at 0.05 mL per nostril). A surrogate ELISA was performed using serum samples to assess the percentage of inhibition against BA.1 on Days 0 and 4 (6 animals/groups) or Day 14 (3 animals/groups) post-challenge. Each dot represents an individual animal, bars depict the median value of the group at the various time points. Horizontal line represents the threshold of 30% inhibition, above which animals are classified as sero-positive against BA.1.

Supplementary Table S1: Experimental design for booster response of monovalent and bivalent CV0501

Group	Animals	1st prime (Day 1) 2nd prime (Day 21)	1st boost (Day 105)	2nd boost (Day 189)	Blood collection schedule
**1**	**Female Wistar rats (N=6)**	0.9% NaCl	0.9% NaCl	0.9% NaCl	Days 21, 42, 77, 105, 133, 161, 189, and 217
**2**	**Female Wistar rats (N=8)**	ancestral N1mΨ 2 µg	ancestral N1mΨ 2 µg	ancestral N1mΨ 2 µg	
**3**		ancestral N1mΨ 8 µg	ancestral N1mΨ 8 µg	ancestral N1mΨ 8 µg	
**4**		ancestral N1mΨ 2 µg	CV0501 2 µg	CV0501 2 µg	
**5**		ancestral N1mΨ 8 µg	CV0501 8 µg	CV0501 8 µg	
**6**		ancestral N1mΨ 2 µg	Bivalent CV0501/ancestral N1mΨ2 µg (total)	Bivalent CV0501/ancestral N1mΨ 2 µg (total)	
**7**		ancestral N1mΨ 8 µg	Bivalent CV0501/ancestral N1mΨ 8 µg (total)	Bivalent CV0501/ancestral N1mΨ 8 µg (total)	
**8**		BA.1 nonclinical N1mΨ 2 µg	CV0501 2 µg	CV0501 2 µg	
**9**		BA.1 nonclinical N1mΨ 8 µg	CV0501 8 µg	CV0501 8 µg	

N1mΨ, N1-methylpseudouridine.

Supplementary Table S2: Antigens included in ACE2 inhibition assay

SARS-CoV-2 variant	Manufacturer	Cat. #	Lot #
Wild type (B1 isolate)	NMI	–	–
Alpha	NMI	–	–
Beta	NMI	–	–
Gamma	NMI	–	–
Delta	NMI	–	–
Omicron BA1 (15 RBD mutations)	Sino Biological	40592-V08H121	LC16FE2107
Omicron BA1 (12 RBD mutations)	Sino Biological	40592-V08H122	LC16MA2204
Omicron BA2	Sino Biological	40592-V08H123	MA16MA1803
Omicron BA2.12.1	Sino Biological	40592-V08H132	MB16MY1181
Omicron BA4	Sino Biological	40592-V08H130	LC16JU1717
Omicron BA5	Sino Biological	40592-V08H131	MA16JL0542

RBD, receptor binding domain; SARS-CoV-2, severe acute respiratory syndrome coronavirus-2.
